# Being a right parent: a narrative review of the theory and practice of parental involvement in sport parenting

**DOI:** 10.3389/fpsyg.2024.1412708

**Published:** 2024-06-07

**Authors:** Chuchen Liu, Fang Zhao, Shujun Nong, Zhiyi Lin

**Affiliations:** ^1^School of Physical Education and Health, Nanning Normal University, Nanning, China; ^2^School of Physical Education and Sport Science, Fujian Normal University, Fuzhou, China

**Keywords:** parental involvement, sport parenting, parent–child interaction, parenting experiences, youth sport

## Abstract

The family is the first classroom for children and adolescents to learn and grow, and parents’ behavior plays an important role in influencing their children’s development, which is also evident in the process of sport participation. The main purpose of this study is to summarise the specific theoretical and practical experiences of parents in sport parenting based on a comprehensive review of the types and functions that constitute parental involvement in sport parenting and the process of their practice. To this end, this study used narrative research as the main research method and searched the literature related to parents’ involvement in parenting through sport using the Web of Science database. Using the theoretical underpinnings of parents’ implementation of sport parenting and their role practice, studies were screened and 39 pieces of literature were finally obtained. The study found that in terms of theoretical underpinnings, the existing types of parental involvement in sport parenting can be broadly categorized into four types: authoritative, authoritarian, permissive and rejecting-neglecting. The functions of parental involvement in sport education have two dimensions: promoting sport development and promoting socialization. Based on a review of their theories, we further summarise and conclude the consequences of action and appropriate practices of parental practices in three scenarios: on the sports field, on the way home and in the private space. It is assumed that parents, when participating in sports parenting, need to: (I) regulate their own behavior in order to avoid psychological pressure on their children due to inappropriate behavior; (II) play different roles at different stages of their children’s sports development; (III) should not put too much pressure on their children’s performance. Based on these reviews of the theory and practice of parental involvement in sport parenting, this study further examines the theoretical limitations of the established research. It is argued that future research should pay attention to the differences between the identities and expectations of parents or children of different genders about their sport parenting, in addition to the differences in parental involvement in sport parenting and different practices in different cultural contexts.

## 1 Introduction

Parental involvement in sport parenting refers to the practice of parents participating in their children’s sport participation as coaches and supporters, and educating their children’s concepts, psychology, and personality through various situations of sport participation in order to promote their socialization process (Krijan et al., 2023). On this basis, combined with the social expectations of parental roles, it is possible that the optimal effect of parental involvement in sport parenting should be the ability to maximize positive effects such as promoting children’s sport participation and enhancing their socialization (Dorsch et al., 2009; Sarı and Bizan, 2022). For example, maximizing children’s enthusiasm for sport and promoting the acquisition of good values.

Currently, there are more studies in the fields of education, psychology and physical education that explore the topic of parental involvement in sport education. Their perspectives are diverse and many results with reference value have been obtained. There are studies from the children’s perspective, discussing the impact of parental behavior on their sport participation process from the children’s perspective, and analyzing what should be done to improve the relationship between parents and children in order to increase the effectiveness of parental intervention in sport parenting (Knight et al., 2010, 2011). However, these perspectives have neglected the role of parents in sport parenting and the important role they play. Subsequently, scholars have focused on the important role of parents in youth sport participation from a parental perspective. For example, Knight and Holt (2013a,b) continued their previous research by investigating parental influences on youth sport through a study of parental performance and child feedback during a tennis match, and suggested that parents could support their children in a more positive way by educating and supporting their children, providing parents with coping strategies, and improving the organizational environment of the sport in which it is played in order to achieve good sport parenting.

Subsequently, some scholars have attempted to further relate parental personality to the effects of sport parenting, developing theoretical models that have the potential to inform subsequent research. For example, Holt and Knight (2014) proposed four types of parental involvement in sport parenting from the dimensions of “response” and “need”, which provided theoretical support for subsequent studies exploring the function of sport parenting. Subsequently, some studies have attempted to use these personality types as a basis for exploring the specific positive effects of parental intervention on children’s sport participation. For example, direct parental support for children’s sport participation (coaching sport techniques, providing sport tactics, etc.) versus indirect support (providing encouragement, providing financial support, etc.), and the positive effects of these supports on children’s sport participation (Coates and Howe, 2023; Rathi et al., 2024). Or, alternatively, to explore the positive effects that parental sport parenting specifically has on a child’s development. For example, improving children’s social skills, learning social norms, and developing good behavior (Kovács et al., 2022). Other studies have looked at parental sport parenting in different settings to discuss the effects of parental sport parenting in different settings. For example, Elliott and Drummond (2017) found that parental misbehavior during a child’s game can produce negative personality traits in the child, such as cowering and fearfulness. And negative parental comments about their child’s training on the way home from training can also have a negative effect on the child’s personality, attitude toward sport, etc. (Tamminen et al., 2022; Thrower et al., 2022). The process of sport parenting in the privacy of the home, in the room, etc., can also be influenced by the way parents practice. Knight et al. (2016), for example, found that parental contributions (including time, money, etc.), which are constantly talked about during meals at home, can create significant psychological stress for children. This results in a dual framework of parental involvement in sports parenting from theory to practice.

However, there are few existing studies that provide a comprehensive overview of the topic. In addition, most of the existing studies discuss the specific effects and rationale of parental involvement in sport parenting only from the perspective of theory or practice. In order to better understand the specific situation of parental involvement in sport parenting, this study attempts to explore the following three sub-questions, namely “What is the theoretical basis of parents’ sport parenting?” “What are the specific functions of their involvement in sport?” and “How do parents implement sport parenting in practice?” Therefore, this study attempts to explore these questions through a narrative review and to identify directions for future research. To provide theoretical support and practical experience for the challenges faced by current research on parental sport education and subsequent practice of parental sport education. And, based on the review of existing findings, it attempts to further suggest the limitations and future research directions in this area.

## 2 Methods

In this study, we are concerned with how parents realize sports parenting. A narrative review will be used to analyses the findings. Even though research conducted through the narrative review method may result in biased findings due to a lack of systematic selection of literature and rigor in the quality of the research (Snilstveit et al., 2012). However, there is merit in this research approach when the purpose of this paper is to illustrate the theoretical and practical experiences of parents involved in sport parenting today (Barker et al., 2023). For example, even if the narrative review does not translate the results of previous studies into general indicators from which scales or rating systems can be generated, as is generally the case with systematic reviews, it can still provide a comprehensive and understandable overview of the state of research on a particular topic (Webster and Watson, 2002; Snilstveit et al., 2012). The narrative approach involves summarizing, comparing, interpreting, and presenting empirical evidence and interview material related to parental sport education (Webster and Watson, 2002). Mainly used to summarise and explain the results of research on a particular issue or topic in order to summarise its theoretical underpinnings or practical experience(Barker et al., 2023).

We used the search platforms Web of Science. All sources were last searched on April 30th, 2024. The development of search terms reflects the understanding of the research themes and issues by all our authors. The main keywords were “family”, “home education”, “parents”, “parenting experiences” and the keywords “sport” and “sport participation” were combined and ordered alternately. The following five keyword combinations were obtained:

1. parental involvement and sport;
2. parenting experiences and sport;
3. family education and sport;
4. parent and sport parenting;
5. family and sport parenting.

In addition, we retrieved the reference lists of some of the review studies to extend the scope of this paper. The types of studies such as newspaper articles and book reviews were subsequently excluded from the scope of this paper (Gunnell et al., 2022).

Subsequently, to ensure the quality of this study, the retrieved literature was screened through the following criteria: (I) Published in the Web of Science Core Collection; (II) Focuses on the role and practice of parents in sport parenting; (III) Explicitly mentions how parents can be involved in their children’s physical activities; (IV) Focus on Parents in Sports Parenting;(V) Did not include the influence of other significant others (e.g., coaches, agents, etc.). After screening 98 papers of high relevance to the study, each author of this study identified the articles to be included in the review by reading them in detail. The articles were further screened by reading them to understand their basic information. After the views of four authors were agreed, 39 articles were finally included in the study. Subsequently, the four authors of this study extracted and organized the information related to this study. First, studies related to parental involvement in sport parenting were intensively read, and the theories used in the literature, the benefits of parental sport parenting for children, and the process of specific sport parenting practices (including sports, children’s information, parents’ information, interaction modes, and places of interactions, etc.) were organized through EXCEL software. Second, similar research findings were summarized and merged. Third, the rationale that can explain these findings is collated from these studies. In this way, the data integrated, analyzed and synthesized support this narrative review.

The keywords were then further condensed into a research question by the four authors based on the literature review: What is the theoretical basis for parental involvement in sport parenting, what are the specific functions of their sport involvement, and how do parents realize sport parenting in practice? Finally, theoretical, and practical exploration of parental sport parenting through narrative review.

## 3 Parent–child interaction: theory and practice of parental involvement in sports parenting

It has been demonstrated that parents play a pivotal role as “both providers of opportunities for youth sport participation and interpreters of the sport experience” (Gould et al., 2006). This implies that parents are not only instrumental in offering opportunities for youth sports but also significantly shape how young individuals perceive their sporting experiences. For example, interpreting failure in competition as a “lack of personal ability” and “better than last time” are two contrasting responses with varying impacts on adolescents’ sports development and socialization. Considering this, scholars have acknowledged that parental involvement in sports parenting should be an integral aspect of adolescent development. It involves a two-way interaction between parent and child, incorporating underlying theories, such as the types and roles of parental involvement in sports parenting, as well as specific practices. This interaction needs to be abstracted, sorted, and organized to provide concrete support for subsequent parental involvement in sports.

### 3.1 A theoretical foundation of parental involvement in sport parenting

**Figure 1:**
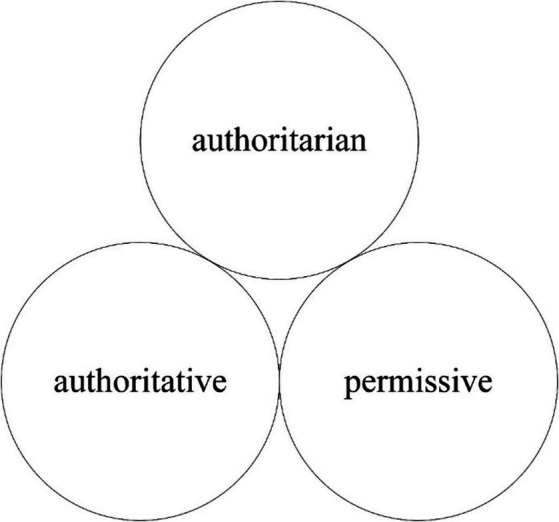
Baumrind’s three types of parental involvement in sport parenting.

Baumrind (1968) introduced three types of parental involvement in sport parenting: authoritarian, permissive, and authoritative, based on parental attitudes and control levels over their adolescents, as depicted in Figure 1. According to this typological analysis, authoritarian parents expect adherence to their interpretation of sports rules, rigidly setting goals for youth sports without providing a clear rationale. Conversely, permissive parents are less likely to demand athletic goals, tolerate youth misbehavior, and use minimal punishment. Authoritative parents strike a balance between the two extremes, determining the training and rest time for their child each day, while also engaging in discussions to explain the reasons behind these demands (Baumrind, 1968).

With the development of the field of parental involvement in sport parenting, some scholars gradually found that the three types of parental involvement in sport parenting proposed by Baumrind were too rough a basis for classification and were not sufficient to exhaust the types of parental involvement in sport parenting. Thus, McCoby (1983) introduced the dimensions of “response” and “demand” into parental involvement in sport parenting, and categorized the authoritative type of parental involvement in sport parenting as high in demand and high in response, and the authoritarian type of parental involvement in sport parenting as high in demand and low in response. Four types of parental involvement were identified: authoritative with high demands and high responses, authoritarian with high demands and low responses, permissive with low demands and high responses, and rejecting-neglecting with low demands and low responses (McCoby, 1983).

**Figure 2:**
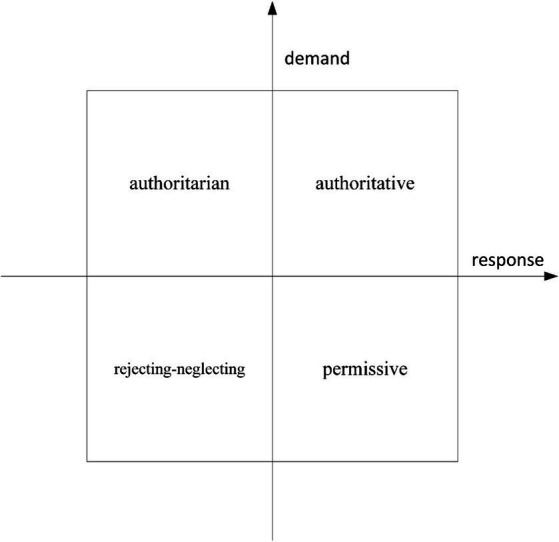
Four types of parental involvement in sport parenting.

Accordingly, the field of sport has referred to this in relation to parental involvement in sport parenting (Holt and Knight, 2014). They have developed four ideal types of parental involvement in sport parenting (shown in Figure 2), drawing on the dimensions of “response” and “demand” as delineated by McCoby et al. Response refers to parental support and feedback to youth participation in sport, including meeting their needs, verbal and operational support, and attention to individual behavior; “demand” refers to parental control of youth participation in sport, mainly including Parental supervision of youth sports training, demands for game performance, and management of daily life.

In contrast to the previous typology of sports parenting, this typology further subdivided permissive parenting into doting parents who pay attention to their children but do not demand it, and neglectful parents who do not care about their children. The former is also referred to as “helicopter” parenting. This means that the parent is like a helicopter, hovering around the adolescent all day and handling all aspects of the child’s athletic participation for him or her. For example, taking care of sports equipment and clothing for outings, carrying bags, doing laundry, cooking, and even tying shoelaces, which leads to a lack of life skills and socialization as the youth grows up (Horn, 2011).

Collectively, studies on parental involvement in sport parenting assert that authoritative parenting is the most effective. Some scholars suggest that further research should aim to establish an ideal parental involvement based on authoritative parenting. This is because, although reciprocal exchanges between authoritative parents and adolescents can be effective, excessive control can undermine adolescents’ internalization of parental values (Lewis, 1981). Additionally, optimal parental involvement in sport parenting styles is not fixed; it varies and is often characterized by multiple situational parenting types (Horn, 2011). Thus, this typology of parental sport participation then provides a theoretical guide for subsequent parental involvement in sport parenting.

### 3.2 Exploring the functions of parental involvement in sport parenting

As mentioned earlier, parental involvement in sport parenting is situational in nature, and academics have found that parental involvement in sport parenting generates functions that arise primarily in both the sport and life arenas, including promoting sport development as well as facilitating socialization.

#### 3.2.1 Parental involvement in sports parenting promotes youth sports

As adolescents mature, their psychological preferences shift toward playing rather than monotonous training solely for skill improvement. Parental involvement in sports parenting has been suggested as a “spice” to keep adolescents engaged in sports (Sarı and Bizan, 2022). By exposing adolescents to different types of sports in the early stages of their participation, parents can identify their children’s athletic talents and stimulate their enthusiasm and interest in sports through guidance and encouragement, providing them with the motivation and energy to participate (Rathi et al., 2024). This practice is more common with authoritative and doting parents who are high in “response” and often reach out for information about their child’s sport (Dorsch et al., 2009). This can be effective in increasing adolescents’ motivation to play sports. One respondent stated:

"...my parents are really supportive and want to make me happy and even come out and run with me as if they can't keep up [with my pace] but they still come out and support me...they keep my passion for the sport alive" (Kramers et al., 2023).

Such parents increase their own sport participation in response to their youth’s interest in the sport, with a view to providing them with more support. In contrast, low “response” parents may ignore their youth’s enthusiasm for sport, leading to premature withdrawal. In terms of providing opportunities for athletic talent development. As individual adolescents’ athletic ability improves, they present a higher demand for coaches’ coaching experience and skills to help them attempt to impact better athletic performance. At this point, parents become progressively less able to coach the skills and abilities needed for athletic progression and begin to shift from direct involvement to indirect support such as financial, time, and logistical support (Coates and Howe, 2023; Rathi et al., 2024). For example, Purchase of nutritious food for children, control of healthy diets, dissemination of nutrition-related knowledge and daily supervision of participation in training programs (Rathi et al., 2024). However, parents play a major role in finding “coaches” for their children who can help them develop their children’s technical talents and playing abilities (Bloom, 1985). As some coaches have stated:

"Some parents have no experience in sports, so they just accompany their children during the morning youth warm-ups. They can't coach their kids without experience and skill, so they hire me to coach" (Burke et al., 2023).

Professionals, like coaches hired by parents, can provide specialized sports training to address young people’s skill deficiencies, offering technical support for effective physical education.

In terms of regulating sport psychology. For adolescents, parents can provide relief from the negative emotions that exist during sports (Kramers et al., 2023). For example, they can provide encouragement during sports losses, keep them from becoming complacent during successes, show them the way during confusion, and strengthen their confidence during wandering. In a study of parental involvement in sports parenting for psychomotor development, one parent made the following action:

“I marked the video of every game he [the child] played, and every time he was feeling a little down and thinking, ‘Oh, maybe I wasn’t that good,’ I put it [the video] on TV and showed him the highlights of what he had done and told him how great he was” (Kramers et al., 2023).

Encouragement and support from close family members can inspire youth to overcome discouragement and cultivate resilient athletic qualities crucial for individual sports development. This is often observed in parental involvement types with high “response,” providing robust support for adolescents’ athletic performance and interest. Conversely, low “response” parental involvement may lead to neglecting a child’s psychological needs in sports, imposing excessive pressure, or ignoring their needs, resulting in “low self-esteem” or “self-pity” on the playing field, which hinders the child’s sports development. Such dynamics are detrimental to the development of individual athletic talents (Kramers et al., 2023).

#### 3.2.2 Parental involvement in sport parenting for youth socialization

Socialization encompasses an individual’s development of personal identity and the acquisition of norms, values, behaviors, and social skills appropriate to their sociocultural setting (Dorsch et al., 2009). Many scholars recognize that sport, as a sociocultural phenomenon, actively nurtures youth socialization through diverse and complex interpersonal interactions during participation. Typically, the family plays an important role in the process of adolescent sport socialization (Dorsch et al., 2009; Fang, 2024). Specifically, parental involvement in physical parenting promotes socialization in terms of transmitting correct social morals, improving mental health, improving interpersonal interactions, pursuing perfection correctly and fostering autonomy (Holt and Knight, 2014; Fang, 2024).

In terms of transmitting correct social morality, parents mainly use their role as companions to transmit correct social concepts to adolescents and change their negative attitudes and perceptions in a timely manner, to promote adolescents’ acquisition of and respect for social norms and awareness of the correct social order. For example, Juntumaa et al. (2005) analyzed the hockey behaviors of 979 parents and their children with different types of sports parenting and found that children with authoritative parents had higher levels of technical and tactical literacy and rule compliance, which led to a greater adherence to the rules and a greater sense of teamwork in their daily lives. Conversely, children with authoritarian parents tend to adopt a win-at-all-costs mentality, directly influencing a utilitarian approach in their daily lives (Juntumaa et al., 2005). Those children under the guidance of rejecting-neglecting or permissive parents have significantly lower levels of technical and tactical skills and adherence to rules than their peers under the guidance of the first two parental types, which will lead to a significant decrease in the enforcement of morals and norms in their daily lives (Juntumaa et al., 2005).

Regarding interpersonal interactions, parents engaged in sport parenting can empower adolescents to improve their social skills through encouragement and practical support, such as introducing them to their friends’ children. Additionally, they can provide tips on elements, norms, and other aspects of interpersonal interactions to enhance their skills (Kovács et al., 2022). This is especially true for children who are introverted due to a congenital disorder or disease, and parents’ use of sports channels to expose them to children with similar experiences can be effective in improving their socialization, such as parents of adolescents with disabilities who state that:

“It’s given him [the disabled child] a whole new group of friends. When we go to disabled teen parties together, we always meet the same group of people. He has gained a lot of friendships there and they are all equal” (Coates and Howe, 2023).

Authoritative parents are effective in developing their children’s interpersonal communication skills with discussions about the use of post-game sports technology and sharing feedback (Kramers et al., 2023). This is markedly different in authoritarian parents, who, due to their own strong will, only demand that their children perform and reject their children’s requests for communication, resulting in their children potentially being less adept at communicating with others (McCoby, 1983). In addition, clubs, camps, and other peer gatherings in which parents enrol their children can provide adolescents with “social practice” outside of school and enhance their social skills.

In terms of pursuing perfection. A lack of parental attention or guidance to the competitive mindset of adolescents may lead to unhealthy perfectionism, or excessive perfectionist strivings (i.e., the pursuit of perfection to achieve high performance standards), and high levels of perfectionist worry (i.e., the desire to achieve high performance standards). Hamachek (1978) argued that authoritarian parents’ high demands and low responses to adolescents tend to trigger this unhealthy perfectionism. Trigger this unhealthy perfectionism, whereas Sapieja et al.'s (2011) study found that authoritative parents would build on their high demands on adolescents by further explaining the reasons for them and providing adequate support to assist their children in developing healthy perfectionism with a proper understanding of themselves. The role of parents in the pursuit of healthy perfectionism is also seen in shaping perceptions of adolescents’ approach to success and failure in sports. As adolescents combat challenges such as failure, fatigue, and injury in sport, parents can impart life lessons, proper morals, and social concepts that buffer their children from negative emotions associated with sport (Côté, 1999). Especially after failures or minor setbacks, parents can provide their children with encouragement or reframe the competitive experience, e.g., when adolescents encounter difficult sports skills to learn or lose in competitions, parents can provide them with the correct conceptualization of learning (step-by-step vs. building on strengths and weaknesses) or interpretation (today’s version of oneself is better than yesterday’s version of oneself), which can lead to the development of correct view of “perfectionism” and provide support and help in facing difficulties in school, work and life (Dorsch et al., 2009).

In terms of fostering autonomy, Holt et al.’s (2009) exploration of parental involvement in sport and the development of autonomy in adolescents found that parental involvement in sport was a form of dedication that demonstrated “parental interest and involvement in their children’s lives.” These parental autonomy behaviors in sports parenting provided models for adolescents to follow in their daily lives. One adolescent commented that:

"They [parents] helped me a lot when I was younger, but they are starting to take a back seat and now I should have everything planned out for me [life, training, and games] like they did" (Burke et al., 2023).

However, it is important to be wary that too much parental involvement can sometimes be counterproductive and prevent children from becoming independent (Kramers et al., 2023). Instead of permissive and rejecting-neglecting parents who simply let their children do whatever they want, authoritative parents who interact with their children may be a good alternative to creating “boundaries” within which their children can make choices, thus fostering their children’s autonomy. In contrast, authoritarian parents have smaller “boundaries” while rejecting-neglecting and permissive parents have larger “boundaries” (Holt et al., 2009).

## 4 Parental role practices and actions in sport parenting reference

The results of the literature review revealed that depending on the situational nature of parental involvement in sport parenting, it was primarily on the field of play, on the way home, and in private spaces. These studies found that positive and negative sport parenting in various scenarios can play a significant role in a child’s motor development and personal growth. Therefore, parents need to control their behaviors and emotions in these scenarios in order to achieve effective sport parenting.

### 4.1 Parental sports parenting practices on the sports field

Whether at practice or during a game, youth often respond to their parents’ on-field behavior. Research suggests that parents focused on winning, punishing, or offering critical feedback tend to appear expressionless or angry in the stands. This can cause adolescents who are focused on this phenomenon to de-embed themselves from the game situation, reflect on the correctness of their actions, and become anxious or fearful. Adolescents who are in this state for an extended period can also have the negative effects extend into their lives, leading to cowering and fearfulness and the development of a negative personality (Elliott and Drummond, 2017). Other scholars have analyzed the impact of parental involvement on adolescents’ performance on the sports playing field and found that such parental performance can have a negative impact on adolescents. One of the interviewees recalled:

"I couldn't even look at my dad during a game, he always had a stern face and I would rather they not be on the golf course because it would just give me a lot of stress" (Burke et al., 2023).

However, if parents can give their children feedback with a nod or a smile when they look, it can be extremely motivating and encouraging (Elliott and Drummond, 2017), as the adolescent respondent stated:

"I like it when my dad smiles at me and gives me a thumbs up, it makes me feel good" (Burke et al., 2023).

This positive feedback is effective in promoting willingness and focus in youth sport participation.

Second, the interactive process between parents and coaches, referees, fourth officials, or other parents can also influence adolescents’ sport performance (Knight and Holt, 2013a). For example, aspects such as parental dissatisfaction with coaching arrangements, resistance to coaching and fourth official calls, or dissatisfaction with other parents’ behavior (Elliott and Drummond, 2017). The field staff commented on this by stating:

"Some parents they like to take care of everything, when you [the child] are on the practice field, they sort all the kid's stuff, make sure they [the child] have all the t-shirts, the gloves, the balls ready to go. But sometimes you see kids don't like that and they chase their parents away" (Burke et al., 2023).

This is mainly because some of the negative behaviors of the parents can create emotions such as shame and embarrassment in the adolescents, which in turn affects their athletic performance. It also makes adolescents accustomed to parental care, which is not conducive to later independent living. And, parents’ language during practices and games-such as yelling in the stands or criticizing their movements and tactics-often causes resentment and embarrassment among adolescents on the field (Dorsch et al., 2015). One adolescent stated:

“Sometimes when the game is going on in general, she [the mother] can be a bit annoying. For example, she would keep yelling at me to get out of my position to get the ball. This is not in line with the coach’s tactics and makes me feel awkward” (Azimi and Tamminen, 2022).

Positive verbal behaviors, on the other hand, are shouting encouragement and cheering loudly, which has the potential to increase adolescents’ feelings of engagement. However, it has also been suggested that this can make some adolescents feel nervous (Bowker et al., 2009).

Beyond parental behavior on the field, negative sports parenting practices often manifest in excessive intervention in adolescents’ athletic decision-making, impacting their development. Parents may insist that their youth participate in higher age groups for greater technical competence. However, this “overlearning” can lead to untimely and early losses, potentially harming psychological perceptions of self-confidence and willingness to play sports. It may even significantly impact adolescents’ psychological development, resulting in introverted tendencies or low self-esteem (Wolfenden and Holt, 2005).

On the other hand, some “demanding” and experienced parents, like authoritative and authoritarian ones, may frequently interfere with coaches’ decisions or change coaches due to personal stereotypes and short-term shortcomings in adolescents’ athletic performance (Holt and Knight, 2014). According to the research, such frequent coaching changes can undermine the authority of the coach’s identity role and the consistency of motor skill acquisition, so that adolescents may develop a contempt for coaches. Moreover, frequent exposure to different sport training styles may overwhelm adolescents. The unfavorable development of athletic talent and motor skill mastery have a greater negative impact (Eliasson, 2018). What’s more, in addition to the mastery of the technical aspects of sports, the influence of coaches on adolescents is also reflected in the quality of sports and technical style shaping. This is the result of long-term moistening of coaches, and frequent coaching changes will undermine this process and have negative impacts on the socialization process of adolescents such as haste and short-sightedness.

Therefore, parents need to focus on playing etiquette on the sports field, avoid providing technical or tactical advice (unless the parent is appropriately knowledgeable and the child has no other coaches), match nonverbal behaviors with supportive language (i.e., remain calm before and after the game rather than yelling), and be consistent throughout the game. Post-game comments focused on the participant’s effort and attitude rather than performance or outcome, providing practical advice to help youth prepare for and recover from the game (Knight et al., 2011).

### 4.2 Parental sports parenting practices on the way back to the house

Sports are often a topic of conversation for parents on their way to and from their youth’s participation in sports training. Parents usually take this opportunity to educate their children. To explore the natural process of this parent–child interaction, a researcher participated in these “private car conversations” by hitchhiking. It was found that the most common conversations in the car were about other roles such as teammates, coaches, and other parents, and consisted primarily of evaluations of other people’s as well as their own children’s performance in competitions, and technical and tactical choices made during games or practices (Thrower et al., 2022). To further explore positive parenting practices on the return journey, Tamminen et al. (2022) counted communication during the sport participation journey and found that they had more non-sport conversations (28.5%) than sport topics (12.9%). In this case, parents’ performance of their children was dominated by praise for listening and paying attention, confidence in their children’s abilities, support for their children’s goals, responsive emotional support, and other aspects of their children’s care. Of course, this does not exclude that the content of their in-car conversations was altered by the researcher’s intervention (Tamminen et al., 2022).

It is important to note that these in-car discussions require parents to be able to use the ‘right’ practices. For example, for evaluating others, parents can communicate with their children objectively and rationally, and promptly resolve their children’s inappropriate evaluations of others and irrationality, or they can implant the seeds of objective thinking for their children by teaching them not to be controlled by their emotions, and even improve their interpersonal skills. For inappropriate technical and tactical choices, take reflective questions to get the child to join in the thinking and discussion. Instead of criticizing them in a violent manner, it will greatly contribute to the child’s intellectual development and personal competence, and will also effectively improve their ability to face and solve difficulties later in life (Sutcliffe et al., 2021).

“In addition, as adolescents grow older, they are more likely to resent in-car lectures by parents or to disapprove of their comments, and to make ‘silent protests’ through silence (59.0% incidence) (Tamminen et al., 2022).”

“Inappropriate conversational styles can even trigger the child to fear the return journey and even refuse to get on the bus due to fear of the return conversation (Tamminen et al., 2022).”

Overall, during the road trip to or back from athletic training, parents communicate their beliefs and values about athletic competition and provide their child with game experience and feedback. This short, enclosed place (in the car) usually provides an environment for parents and children to communicate openly, making the child more receptive to parental education, but it is also important to be mindful of the child’s feedback and attitudes, so as not to provoke rebelliousness, which can lead to counterproductive education (Holt and Knight, 2014). For this reason, parents need to consciously establish ways to communicate with their children, to understand their children’s state of mind and current thoughts, and to respond in a timely manner (Coates and Howe, 2023). In addition, parents also need to pay attention to the content of communication: communication between parents and children should be centered on the child’s attitude and effort in sports training and competition, rather than blaming and criticizing the child based on results and talent, so that the child does not become resistant to communication on the road (Gould et al., 2016). Moreover, reflective questions can be used to stimulate introspection and thinking in adolescents during the road trip, e.g., “What would you do if you were given another chance?” to motivate children’s intrinsic motivation for sport participation and to develop good reflective habits.

### 4.3 Parental sports parenting practices in private spaces

As parents are the guardians of their youth, the common areas of the home or the private spaces of the youth’s room can provide interactive environments for sports parenting beyond the playing field. Adolescents present different mindsets in different arenas, which can result in very different parental responses. For example, if they appear “weak” and “timid” in front of their parents, parents can provide unconditional love, encouragement, and praise, as well as other forms of emotional, tangible, or intangible information support. These positive parental practices may respond favorably to their life responses, for example, through an inclusive upbringing that allows them to maintain positive emotions such as confidence, friendliness, and optimism in their future work and schooling and to be protected from negative emotions (Knight et al., 2010). One adolescent, when frustrated, stated:

"The most important thing for me was that they [parents] made me believe that I was loved, even if the golf [game] wasn't as good [as the scores] ...they cared about the golf but they cared more about me, and yeah, that made me feel great...that made me more comfortable. I know that no matter what happens to me, when I come home, I'm still loved" (Burke et al., 2023).

However, if parents are overly focused on athletic performance rather than their youth’s growth in sports, this may intentionally or unintentionally create psychological stress for them. This leads to a loss of confidence in their athletic training or competition, and may even affect their future growth by making them feel anxious or depressed (Knight et al., 2016). For example, aspects such as constantly talking about parents’ financial contributions and time commitments at home or even at dinner; having unrealistic expectations of athletic performance; and regularly criticizing their child’s performance on the athletic field (Knight et al., 2016). If parents view the time and resources they give to their children’s participation in sports, such as driving them to and from their games, watching them play, and providing them with financial support, as sacrifices they make themselves, it can similarly have a significant impact on causing psychological stress in youth (Dorsch et al., 2009).

Additionally, studies have shown a strong correlation between the frequency of sports topics discussed in private spaces and adolescents’ psychology and age (Kovács et al., 2022). As motor skills grow, the psychological pressure on parents increases due to higher financial expenses. This stress is often transferred to adolescents in private space exchanges and becomes a shared stressor (Kovács et al., 2022). For this reason, there have been calls from researchers that this should be taken seriously to increase parental involvement and the effects produced by their practices to avoid counterproductive effects (Knight et al., 2016). Parents need to work with a sport psychologist to learn to cope with issues arising from their adolescent’s psychological and age-related development or to improve relevant psychological skills to help them make timely adjustments to their own behaviors and strategies in sport parenting (Knight and Holt, 2013b). For example, when children are not awakening to the desire to win or lose in the early stages of sport participation, it is important not to overemphasize winning and performance at home or in other private spaces. Rather, focus on whether they show interest in the program in their daily lives and the benefits of the sport to their individual growth (Fry and Duda, 1997). And it is important to be attentive to the child’s view of self in these private spaces and to help them adjust to poor training results, lost competitions, and so on.

### 4.4 Being a right parent: improving the effectiveness of sports parenting

In summary, existing research has confirmed that parental involvement in sports parenting has a key role in the growth and development of adolescents, which requires them to regulate their behavior in all contexts of the participation process, such as the sports field, the playing field, and the private space(Mallett et al., 2024).

Parents also need to find an appropriate level of involvement, as they play different roles at different stages of their children’s lives. The stage of youth sports development is closely related to parental involvement in sports parenting practices and should also have different involvement practices (Rathi et al., 2024). For example, as a child’s motor skills develop and parents are no longer able to coach, they may need to redefine their role and move to a supportive role, beginning to partner with the coach in education. It is important not to intervene blindly based on one’s own ‘experience’ with the young person, as this can lead to undesirable consequences (Mallett et al., 2024). In addition, parents should continually adapt their child’s role by encouraging them to try different programmes at different stages. They should even support them when they make mistakes and help them to adapt and develop through unconditional support. However, children should not be too actively involved in competitions or training that are out of their age group, such as expecting them to excel in older age groups or to train or play on full-size courts and show immediate improvement. Or perhaps they change coaches cautiously, trusting their coach to deliver long-term results rather than overreacting to short-term wins and losses. On this basis, parents can provide effective intervention and guidance in their children’s sports participation and get sports parenting right.

## 5 Conclusion

The important influence of parents in intervening in their children’s sport participation was reflected in a review and summary of 39 papers included in the study. Most of the published research suggests that different types of parents in sport parenting can have a positive effect on children’s sport participation in terms of effective financial and emotional support, or a negative effect in terms of children’s fear of competition and reluctance to participate in training, because of their attitudes and behaviors toward their children. For example, authoritative parents may have a positive effect such as fostering correct perceptions and personal behaviors of sport participation in their children because of their more impartial attitude and respect for their children’s wishes. On the other hand, authoritarian parents will have a negative effect on their children’s participation because of their authoritarian attitude and disregard for their children’s ideas, which may lead to the development of negative sports emotions and habits, and a fear of sports training. In addition, this study explores how parents should parent in sport through the specific practices of three different scenarios: the ‘sports field’, ‘on the way home’ and ‘private space’. For example, on the sports field, parents should pay attention to their children’s mental state and provide them with timely encouragement and support instead of criticizing and blaming them for their performance, and so on. All of these provide theoretical and practical guidance for parents to achieve optimal parenting outcomes through sport activities.

It should be noted, however, that since most of the existing research has focused on developed countries such as Europe and the United States, few scholars have focused their research perspectives on the diverse phenomena that exist in different cultural settings such as Asia and Africa. As a result, further research is needed to complement the parental sport parenting practices identified in this study. On the one hand, it is necessary to consider the differences in children’s personal perceptions, mental capacity, and physical fitness in different cultural contexts. On the other hand, the role of parents also varies considerably between countries in terms of culture and economic level. Under the combined influence of these two situations, we need to consider in the future how parental involvement in sport parenting may differ in the same situation under the influence of different socio-cultural factors in different regions. For example, the different effects of the same stressors on parents and children in different cultural contexts. Or the differences in parents’ expectations of their children’s sport participation in these different cultural contexts.

It is also important to note that “parent” is not a complete entity. The two roles of father and mother have different influences on the promotion of children’s participation in sport. This influence is based on different gender constructions, where individuals with different gender identities may have different role identities and carry different social expectations. For example, in the current social culture, the role of the father or mother should be more important in the implementation of sport parental activities. At the same time, for children of different sexes, there seems to be a need to consider their gender differences and the resulting differences in personality and needs. Therefore, future research should also consider the different combinations of sport parenting between parents and children from different gender perspectives.

## Author contributions

CL: Conceptualization, Data curation, Methodology, Writing – original draft, Writing – review & editing. FZ: Methodology, Supervision, Visualization, Writing – review & editing. SN: Conceptualization, Methodology, Writing – original draft, Writing – review & editing. ZL: Conceptualization, Data curation, Methodology, Supervision, Validation, Writing – original draft, Writing – review & editing.
